# Wideband Self-Calibration Method of Inductive CTs and Verification of Determined Values of Current and Phase Errors at Harmonics for Transformation of Distorted Current

**DOI:** 10.3390/s20082167

**Published:** 2020-04-11

**Authors:** Ernest Stano, Michal Kaczmarek

**Affiliations:** Institute of Mechatronics and Information Systems, Lodz University of Technology, Stefanowskiego 18/22 St, 90-924 Lodz, Poland; michal.kaczmarek@p.lodz.pl

**Keywords:** inductive current transformer, current and phase errors, harmonics, distorted current, power quality, composite error, vectorial diagram, equivalent circuit, ampere-turns

## Abstract

Self-calibration of a designed wideband inductive current transformer (CT) was carried out in the ampere-turns condition. This method does not require a reference transducer. The values of current and phase errors at the harmonics of frequencies from 100 Hz to 5 kHz were determined for the distorted primary current of the rated main frequency equal to 50 Hz. These results were verified based on the comparison of values measured between two CTs and calculated as the difference between values obtained from their calibration. Moreover, from vectorial diagrams drawn for transformation of the higher harmonics, the source of the change in the values of current and phase errors with frequency is explained. Furthermore, the method for calculation of the values of the corresponding harmonics of the current associated with the active power losses in the core and the magnetization current is presented.

## 1. Introduction

The advantages of inductive current transformers (CTs) are reliability, long lifetime with the provided accuracy class, and low cost with no requirement of additional power supply. Due to the distortion of voltages and currents of the power network, the significance of the power quality increases. Therefore, inductive instrument transformers should ensure accurate transformation of higher harmonics [1]. However, none of the IEC\IEEE standards specify requirements and methods for its evaluation. The standard IEC 61869-6 defines only limiting values of the ratio and phase errors at harmonics for low-power instrument transformers, i.e., active or passive electronic circuits designated to voltage or current transformation [2]. The specific standard refers to passive Rogowski coils and passive current to voltage transducers [3]. Verification steps of instrument transformers and protocols consisting of the most important parameters of the tested power quality instruments were proposed in [4]. Determination of the voltage and phase errors of inductive voltage transformers (VTs) requires usage of a wideband reference voltage divider [5,6,7]. The measuring system may consist of a power network analyzer or digital power meter enabling computation of the FFT spectrum of the measured distorted voltage [8,9]. A similar approach may be used for evaluation of the transformation accuracy of harmonics of the distorted current by inductive CTs [10,11,12]. The paper [10] presents the application of the directly measured values of the composite error that corresponds to the maximum values of the ratio and phase errors for characterization of the wideband accuracy of inductive CTs. This ensures high accuracy of the obtained results with low measuring apparatus costs. To perform the measurements, an additional primary winding was made. In order to obtain the rated ampere-turns state, its numbers of turns must be equal to the rated current ratio of the tested CT. Therefore, the values of the primary and secondary currents of the tested CT are equal to each other. Such an approach is limited to window-type CTs. Characterization of the transformation accuracy of distorted current may be performed with the fundamental frequency and one higher harmonic [2,10,11]. The article [12] provides a proposition of evaluation criteria for testing the transformation accuracy of inductive CTs for the harmonics of distorted current. This solution, irrespective of the types of primary connection and the primary converter, allows evaluation of the accuracy class extension of CTs designed for power quality measurements. In the proposed measuring system, a flux-gate technology wideband current transducer is used for reference. Obtained results of the accuracy tests of wideband transformation indicate the same requirements for current errors at harmonics, as defined in the standard IEC 61869-2 for sinusoidal current and a given accuracy class [13]. This mainly results from low-frequency-dependent properties of the magnetic core [14,15]. Moreover, transformation of distorted currents may cause its saturation [16,17,18,19]. The method presented in paper [16] helps in detecting the exact saturation period, and, thus, signal reconstruction or time delay logic can be applied to avoid the error-in-estimation process. Furthermore, the usage proposed in paper [18] for the variational mode decomposition of secondary current allows conditions of magnetic core saturation to be defined. Calibration of CTs during the transformation of 50\60 Hz sinusoidal current requires application of the reference CT and comparator for secondary currents [13,20]. Other methods of accuracy verification of the tested CTs in these cases are based on usage of the measuring systems containing a Rogowski coil or flux-gate technology wideband current transducer [2,12,21]. In such systems, a digitizer with digital signals proportional to the phase and ratio errors of the tested CT may be implemented to increase the accuracy [22]. The device utilizes a dual-slope A/D converter and implements digital computation of errors. Therefore, the digitizer maintains its accuracy over a wide frequency range and, after implementation, the FFT algorithm can also be used for defining errors of the CT during transformation of the distorted currents. The values of current and phase errors of the harmonics transformation by the tested CTs can also be determined with the two-channel data acquisition board presented in [23,24]. All presented methods require a reference device. Therefore, to simplify the measuring circuit and decrease its cost with a simultaneous increase in accuracy, a self-calibration method is proposed in this paper. The values of current and phase errors at harmonics during the transformation of distorted current by inductive CTs are determined in the ampere-turns condition.

A wideband inductive CT with a rated primary current rms value equal to 300 A and rated secondary currents rms values equal to 5 and 1 A is designed for harmonics transformation up to and including the 100th order with the main frequency of distorted current equal to 50 Hz. The obtained wideband accuracy class for its rated load of each of the secondary windings equal to 2.5 W is 0.05 as for the transformation of sinusoidal current in accordance with the standard IEC 61869-2 [12]. The maximum values of current and phase errors are presented in Table 1. In the evaluation method to obtain the equality of ampere-turns additional primary windings with 60 turns or 300 turns is used instead of the primary wire. This self-calibration method is verified through comparison of the results obtained between two CTs tested in the ampere-turns conditions. Furthermore, analysis of the determined values of current and phase errors indicate that if high accuracy is ensured for the main component of distorted current, it may also be obtained for higher harmonics. As modern magnetic materials are characterized by high wideband magnetic permeability, and low power losses are commonly used, the most important is the negligible self-distortion of secondary current caused by nonlinearity of the magnetic core. Moreover, vectorial diagrams are used to assess parameters of the equivalent circuit of the designed wideband inductive CT during transformation of selected higher harmonics of distorted current. The possibility of application as a reference CT of a typical 0.2 accuracy class window type unit designed for transformation of the sinusoidal current of frequency 50 Hz is also analyzed.

## 2. Wideband Self-Calibration of Inductive CTs with Application of Additional Primary Winding

**Figure 1:**
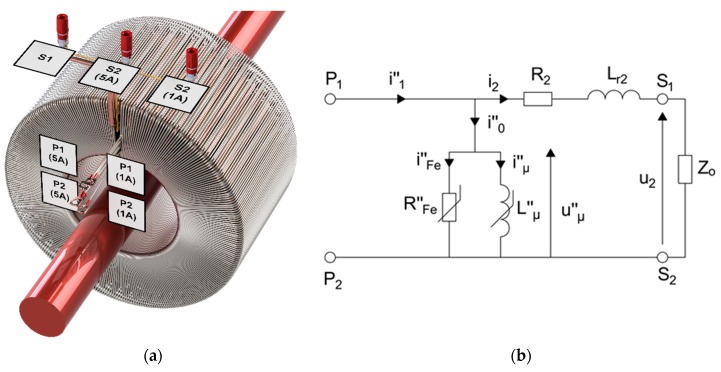
(**a**) 3D view of designed wideband current transformer (CT), (**b**) equivalent circuit of inductive CT.

The magnetic core of the developed wideband inductive CT presented in Figure 1a is made of Ni80Fe20 tape [14]. Its quality preliminarily determines the current and voltage errors of the transformation of sinusoidal, as well as distorted, current. This is due to the fact that the instantaneous value of the primary current is equal to the sum of the instantaneous value of the secondary current multiplied by the rated turns ratio and instantaneous value of exciting current of the magnetic core (Figure 1b).

In Figure 1a,b, the following notations are used: (symbol ‘‘ means values converted to the secondary side of CT): *S1*—common terminal of the secondary windings, *S2(1\5 A)*—terminal of the 1\5 A secondary winding, *P1\2(1\5 A)*—terminals of the additional primary winding for testing the accuracy of transformation to the rated secondary current equal to 1\5 A, *i^″^_0\1\2_*—instantaneous value of the exciting\primary\secondary current, *R^″^_Fe_*\*i^″^_Fe_*—resistance\current associated with the active power losses, *L^″^_μ_*\*i^″^_μ_*—main inductance\magnetization current, u_2_—instantaneous value of the secondary voltage, *L_r2_—*leakage inductance of the secondary winding, *Z_O_*—impedance of the load. The value of the active power losses results from the hysteresis and the eddy current losses in the core. The magnetization current is responsible for generation of the magnetic flux. 

**Figure 2:**
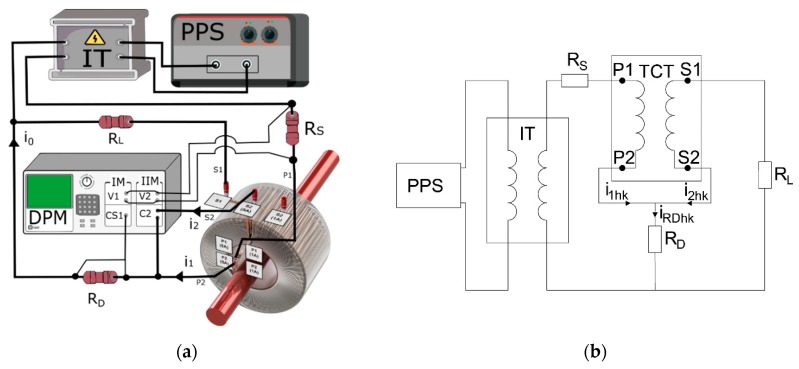
(**a**) Measuring circuit used to determine the values of current and phase errors of higher harmonics transformation in the condition of equivalent ampere-turns, (**b**) simplified wiring diagram.

In the evaluation procedure, the wideband transformation accuracy of the distorted primary current by inductive CTs comprising the main frequency harmonic (50 Hz) and one higher harmonic of orders from 2nd to 100th is used. Although, the most significant self-distortion of the inductive CT secondary current occurs for the highest obtained magnetic flux density in its magnetic core. This may result from its secondary winding load and primary current rms value, as well as its shape, depending on the higher harmonics content and their phase angle in relation to the main component. The primary objective of the research is the evaluation of the transformation accuracy of harmonics of the distorted current by the developed wideband inductive CT to both secondary currents. Moreover, the wideband transformation accuracy of window-type inductive CTs with rated current ratios equal to 100\5 or 1 A and 300\5 or 1 A designed for transformation of the sinusoidal current of frequency 50 Hz is also tested. Rated loads of their secondary windings are equal to 2.5 VA. However, during the tests, purely resistive loads are used to not additionally increase the secondary voltage and magnetic flux density for transformation of higher harmonics of the distorted primary current. The measuring circuit to determine the values of the current and phase errors of higher harmonics transformation in the condition of equivalent ampere-turns is presented in Figure 2a.

In Figure 2a,b, the following notations are used: *PPS*—programmable power source, *IT*—insulation transformer, *TCT*—tested CT, *R_D_*—10 Ω current shunt for measurement of differential current, *DPM*—digital power meter, *R_L_*—load of tested CT, *R_S_*—0.1\1 Ω current shunt for measurement of primary current 5\1 A.

The differential connection (Figure 2a) is made in accordance with standard IEC 61869-2 [13]. However, it is used here to determine the composite error of measuring CTs. A digital power meter with DFT function enables the determination of its particular higher harmonics. The current shunt *R_D_* with a resistance equal to 10 Ω is used to measure differential current in the connection wire between additional primary and secondary windings of the tested CT. The measuring circuit is supplied by a programmable power voltage source through an insulating transformer to enable generation of distorted primary current. A digital power meter is used to measure simultaneously rms values of the harmonics of voltages from *R_S_* and *R_D_* current shunts and the phase angles between them. The rms value of the *hk* harmonic of the reference primary current in the condition of ampere-turns or secondary current of the reference CT is determined from following equation:

(1) $$I_{1hk}=\frac{U_{RShk}}{R_{S}}.$$

*I_1hk_*—the rms value of the *hk* harmonic of the primary current, *U_RSkh_*—the rms value of the *hk* harmonic of voltage on current shunt R_S_, where its resistance is equal to 0.1 Ω for the rated additional primary winding, and its secondary currents are equal to 5 A and 1 Ω for the rated currents during the test equal to 1 A.

The percentage value of the *hk* harmonic of the composite error in relation to its rms values in the distorted primary current is determined from equation [12]:

(2) $$\varepsilon_{\%Ihk}=\frac{R_{S}\cdot U_{RDhk}}{R_{D}\cdot U_{RShk}}\cdot 100\%.$$

*U_RDkh_*—the rms value of the *hk* harmonic of voltage on current shunt R_D_ of resistance equal to 10 Ω.

The rms values of harmonics of the secondary current are determined from the following relationship [12]:

(3) $$I_{2hk}=\sqrt{{\left(\frac{U_{\mathrm{RS}hk}}{k}\right)}^{2}+{\left(\frac{\;U_{RDhk}}{10}\right)}^{2}-2\cdot \frac{U_{\mathrm{RS}hk}}{k}\cdot \frac{\;U_{RDhk}}{10}\cdot \cos_{hk}}.$$

*U_RSkh_*—rms value of the *hk* harmonic of voltage on current shunt R_S_ with resistance equal to 1 or 0.1 Ω, *φ_hk_*—phase angle between *hk* harmonic of voltages from resistors *R_S_* and *R_D_*.

The current error of transformation of the *hk* higher harmonic of the distorted current by the tested CT if the primary and secondary current are equal is determined from the following equation [12]:

(4) $$\Delta I_{hk}=\frac{I_{2hk}-I_{1hk}}{I_{1hk}}\cdot 100\%.$$

*I_2hk_*—the rms value of the *hk* harmonic of the secondary current of the tested CT.

Determined *hk* values of composite and current errors are used to calculate the value of the phase error of transformation of the higher harmonic of the distorted current by the tested CT [12]:

(5) $$\delta_{hk}=\arcsin\left(\frac{\sqrt{\varepsilon_{\%Ihk}^{2}-\Delta I_{hk}^{2}}}{100\%}\right).$$

When the phase angle between the *hk* harmonic of voltages from current shunts *R_s_* and *R_D_* is between 0° and 180°, the phase error is positive, and negative in the other case. The sign of the current error is positive when the phase angle is between 90° and 270°, and negative in the other case [12].

**Figure 3:**
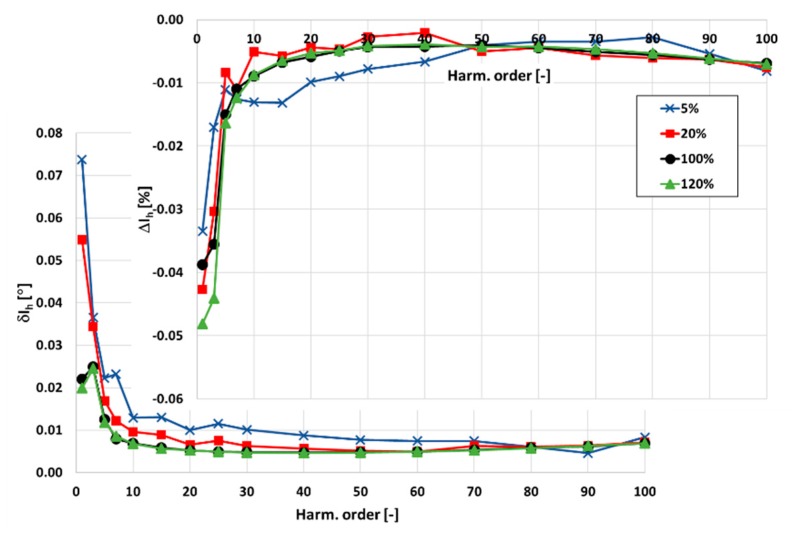
The values of current and phase errors at harmonics of the developed wideband CT for secondary current 1 A, rated load, and 5%, 20%, 100%, 120% of rated primary current.

**Figure 4:**
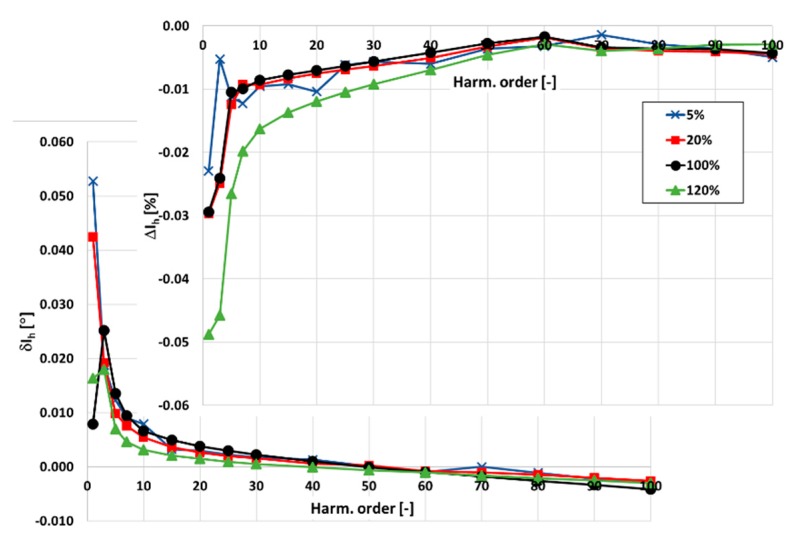
The values of current and phase errors at harmonics of the designed wideband CT for secondary current 5 A, rated load, and 5%, 20%, 100%, 120% of rated primary current.

In Figure 3 and Figure 4, the values of current and phase errors of the transformation of harmonics of the distorted primary current in the range of frequencies from 100 Hz to 5 kHz by the developed wideband inductive CT are presented. The measurements were made separately for secondary windings where the rated secondary current was equal to 1 and 5 A with their rated load when the distorted primary current was equal to 5%, 20%, 100%, and 120% of its rated rms value.

The accuracy class of the tested inductive CT for transformation of the sinusoidal current is 0.05. It results from the 10 times smaller limits presented in Table 1 for the values of current and phase errors defined in the standard IEC 61869-2 for the 0.5 accuracy class. 

The same limits may be used for evaluation of the wideband accuracy class. Due to the self-generation of the 3rd harmonic only, the determined values of current error for the 1 and 5 A secondary current are close to this value. In Figure 4, the values of current and phase errors decrease along with the order of the transformed higher harmonic. This results from the decrease with frequency of the values of the current associated with the active power losses in the core and the magnetization current. In the first case, it is caused by the decrease in magnetic flux density with frequency. In the second case, it is caused by the lower decrease in reactance of the magnetic core made of the Ni80Fe20 tape due to the decrease in its magnetic permeability than its increase with frequency.

**Figure 5:**
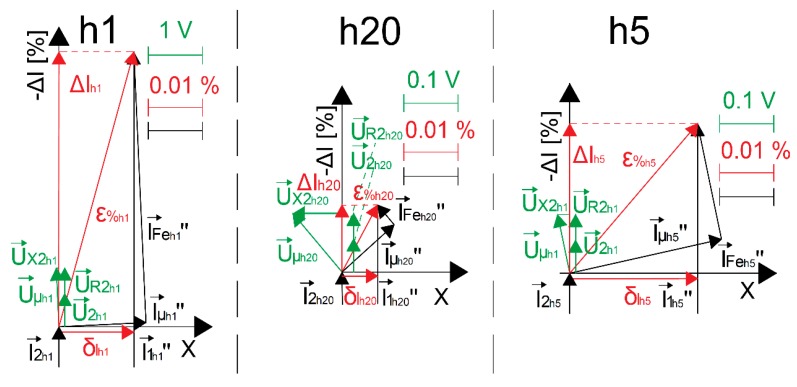
Vectorial diagrams for transformation of the 1st (h1), 5th (h5), and 20th (h20) harmonics of distorted primary current by designed CT (X = sinδ_hk_⋅100%).

On the basis of the determined values of current and phase errors for transformation by the developed wideband CT of the 1st, 5th, and 20th harmonics, the vectorial diagrams presented in Figure 5 may be drawn. The values are determined for the equivalent circuit from Figure 1b in conditions of the rated load and rms value of the secondary current equal to 5 A. The value of its reactance for the 50 Hz component of distorted current due to the evenly wound wires on the toroidal core is adopted as 0.1 of the measured value of resistance for DC.

In Figure 5, the following notations are used: (vectors of the currents and phasors of errors are expressed in [%] of the *hk* harmonic of primary current, vectors of voltages are expressed in [V], symbol ‘‘ means values converted to the secondary side of CT): $\vec{I}$*″_1hk_*—vector of the *hk* harmonic of the primary current, $\vec{I}$*_2hk_*—vector of the *hk* harmonic of the secondary current, $\vec{I}$*″_Fehk_*—vector of the *hk* harmonic of the current associated with the active power losses in the core, $\vec{I}$*″_μhk_*—vector of the *hk* harmonic of the magnetization current of the core, $\vec{U}$*_2hk_*—vector of the *hk* harmonic of the secondary voltage, $\vec{U}$*_μhk_*—vector of the *hk* harmonic of the voltage in the parallel-connected resistance representing core losses and the mutual inductance of windings, $\vec{U}$*_R2hk_*—vector of the *hk* harmonic of the voltage on resistance of the secondary winding, $\vec{U}$*_X2hk_*—vector of the *hk* harmonic of the voltage on reactance of the secondary winding, ΔI_hk_—phasor of the current error of the *hk* harmonic, δI_hk_—phasor of the phase error of the *hk* harmonic converted as *sinδ_hk_⋅100%*, ε_%hk_—phasor of the composite error of the *hk* harmonic.

The vector $\vec{I}$*″_μhk_* is determined geometrically as perpendicular to the vector $\vec{U}$*_μhk_*, and its length results from perpendicular vector $\vec{I}$*″_Fehk_* that ends in a point resulting from determined values of current and phase errors of the transformation of the *hk* harmonic. Presented in Figure 5, vectorial diagrams allow us to draw the conclusion that both current and phase errors decrease with frequency due to the fact that the values of current associated with the active power losses in the core and magnetization current of the core decrease with their frequency. Determined values of the parameters of the equivalent circuit from Figure 1b for the 1st, 5th, and 20th harmonics are presented in Table 2.

Vectorial diagrams for transformation of the 1st (h1), 5th (h5), and 20th (h20) harmonics of the distorted primary current by the inductive wideband CT enable us to calculate, as presented in Table 2, values of the hk harmonics of the current associated with the active power losses in the core and the magnetization current. Then, the values of resistance representing the core losses and mutual inductance of windings for the *hk* harmonics may be determined from the following equations:

(6) $$L_{\mu hk}^{\prime \prime }=\frac{U_{\mu hk}}{I_{\mu hk}^{\prime \prime }\cdot 2\cdot \pi \cdot f_{hk}}$$

(7) $$R_{Fehk}^{\prime \prime }=\frac{U_{\mu hk}}{I_{Fehk}^{\prime \prime }}$$

The vectorial diagram and calculated increase in value of the mutual reactance of windings for the 5th harmonic (Table 2) justify the temporary increase in the transformed higher harmonic of the phase error value with frequency in relation to the main component of distorted current.

**Figure 6:**
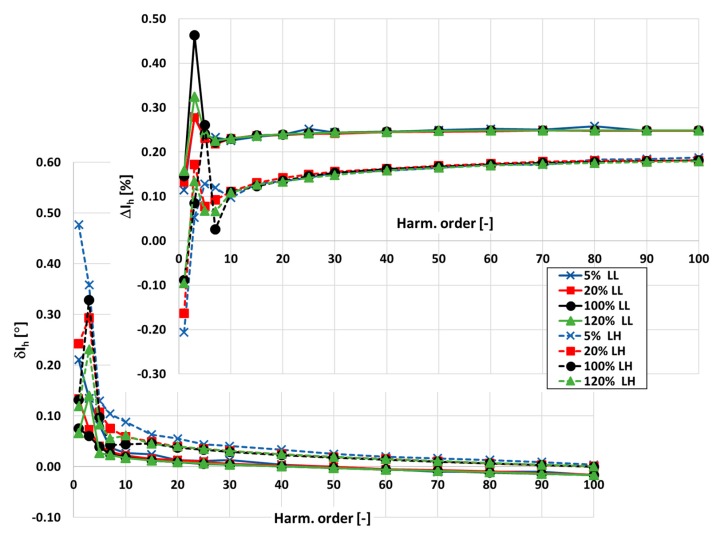
The values of the current and phase errors at harmonics of the 100\1 A inductive CT for 25% and 100% of the rated load of the secondary windings when the distorted primary current was equal to 5%, 20%, 100%, and 120% of its rated value.

In Figure 6, the values of current and phase errors of the transformation of harmonics of the distorted primary current in the range of frequencies from 50 Hz to 5 kHz by the 100\1 A inductive CT are presented. The measurements were made for 25% and 100% of the rated load of the secondary windings when the distorted primary current was equal to 5%, 20%, 100%, and 120% of its rated rms value.

In Figure 6, the following notations are used: 5%\20%\100%\120% LL (load low)\LH (load high)—results curve for a given percentage of rated primary current and 25%\100% of rated load of the secondary winding.

The accuracy class of the tested CT for transformation of the sinusoidal current of frequency 50 Hz is equal to 0.2 [12]. Used by the manufacturer, the turns correction makes the value of the current error significantly positive even for conditions of the rated load of the secondary winding. This is due to the decrease with frequency in the transformed higher harmonic values of current associated with the active power losses in the core and the magnetization current. Its wideband accuracy is additionally conditioned by the self-generation of 3rd and 5th harmonics. The wideband accuracy class of the tested inductive CT for transformation of the higher harmonics of distorted current is equal to 0.5 (if the limits for the values of current and phase errors defined in the standard IEC 61869-2 are used). The accuracy class 0.2, excluding self-generated 3rd and 5th harmonics, may be obtained only for the rated load of the secondary winding. In the case of this tested CT with a rated secondary current value equal to 1 A, the increase in its distortion due to the nonlinearity of the magnetic core with the value of the connected resistance in the secondary winding is negligible. However, for this condition of operation, the change in values of the current and phase errors with frequency of the transformed higher harmonic is much greater.

**Figure 7:**
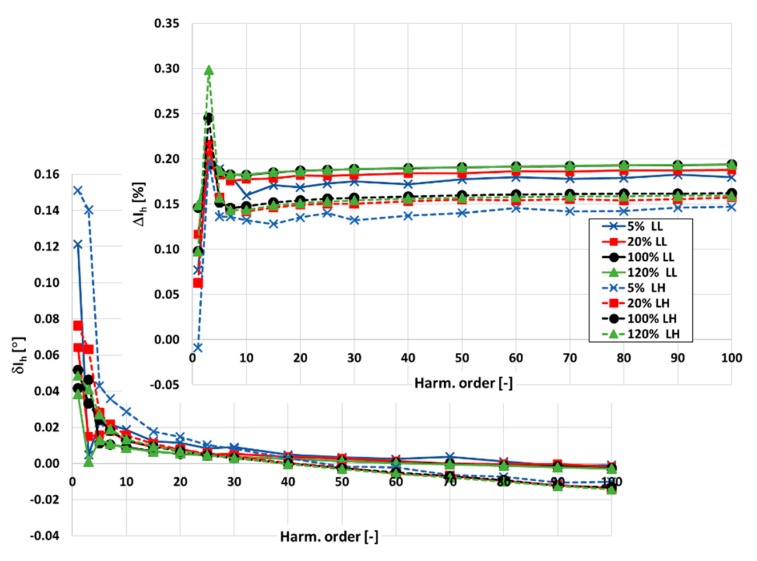
The values of current and phase errors at harmonics of the 300\5 A inductive CT for 25% and 100% of the rated load of the secondary windings when the distorted primary current was equal to 5%, 20%, 100%, and 120% of its rated value.

In Figure 7, values of the current and phase errors of the transformation of harmonics of the distorted primary current in the range of frequencies from 50 Hz to 5 kHz by the 300\5 A inductive CT are presented. The measurements were made for 25% and 100% of the rated load of the secondary windings when the distorted primary current was equal to 5%, 20%, 100%, and 120% of its rated rms value.

The notations used in Figure 7 are same as described for Figure 6.

The accuracy class of the tested CT for transformation of the sinusoidal current of frequency 50 Hz is equal to 0.2 [13]. Its wideband accuracy is deteriorated to 0.5 by self-generation of the 3rd harmonic. In the case of this tested CT with a rated secondary current value equal to 5 A, the increase in its distortion due to the nonlinearity of the magnetic core with the value of connected resistance in the secondary winding is significant. However, for this condition of operation, the change in values of current and phase errors with the frequency of the transformed higher harmonic is negligible.

## 3. Verification of Determined Values of Current and Phase Errors at Harmonics

**Figure 8:**
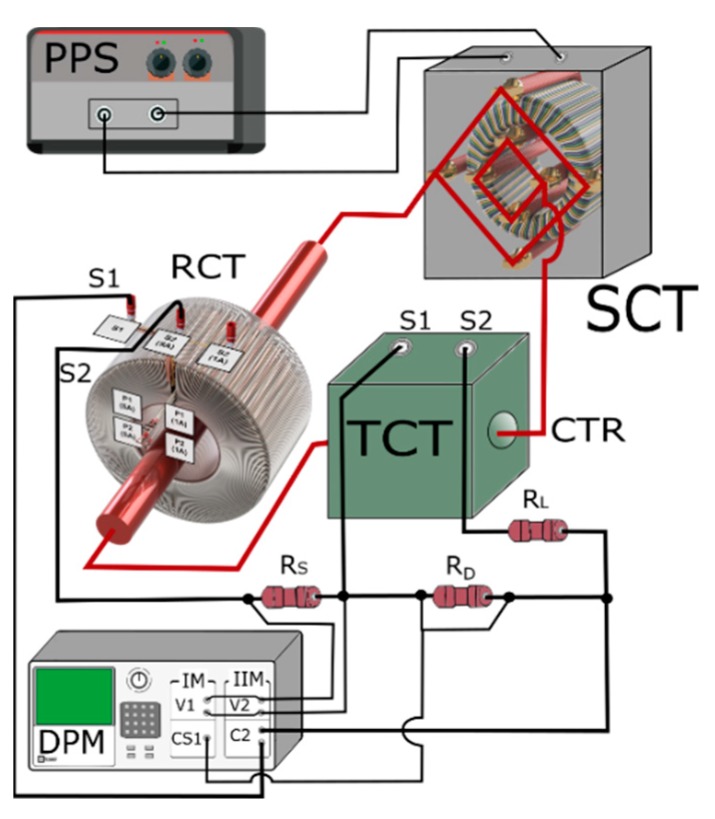
Measuring circuit used to determine the values of current and phase errors of the higher harmonics transformation between two CTs.

The measuring circuit used to determine the values of current and phase errors of the higher harmonics transformation between two CTs is presented in Figure 8.

The notations are used as in Figure 2, additionally: *SCT—*step-up current transformer, *RCT*—CT used as reference of primary current, *CTR*—high current track.

The step-up current transformer was previously tested during transformation of the distorted currents. In the paper [25], it was demonstrated that it is capable of wideband operation and shows no resonance in the tested frequency range of higher harmonics up to 5 kHz. Equations (1) to (5) are further used to determine values of the current and phase error of the transformation of harmonics of the distorted current. 

In accordance with the information provided by the manufacturer, the tolerance of resistance of the used current shunts is equal to ±1% for 0.1 Ω, ±0.02% for 1 Ω, and ±0.01% for 10 Ω. The value of their inductance is below 0.08 μF. Therefore, calculated from Equations (8) and (9), the measurement errors of the 50 Hz frequency main component of the distorted current and its phase caused by current shunts are respectively equal to ±1%\±0.02° for 0.1 Ω, ±0.02%\±0.02° for 1 Ω, and ±0.01%\±0.01° for 10 Ω.

(8) $$\;\Delta U_{Rhk}=\Delta U_{RDC}+\frac{Z-R}{R}\cdot 100\%$$

(9) $$\delta_{Rhk}=\tan^{-1}\left(\frac{2\cdot \pi \cdot f\cdot L}{R}\right)$$

Δ*U_RDC_*—tolerance of resistance of used current shunts, *Z*—impedance of used current shunts for a given frequency of higher harmonics, R—the value of DC resistance of current shunt, f—frequency of higher harmonic, L—the value of the inductance of a given current shunt (0.08 μF).

Calculated from Equations (8) and (9), measurement errors of the 100th higher harmonic (5 kHz) of distorted current and its phase caused by current shunts are respectively equal to ±1.05%\±1.5° for 0.1 Ω, ±0.02%\±0.15° for 1 Ω, and ±0.01%\±0.01° for 10 Ω. The measurement errors of rms values of the harmonics of voltage by the used digital power meter are equal to ±0.2% for 50 Hz and ±0.75% for 5 kHz. The measurement errors of phase displacement between the harmonics of voltages (currents are measured through voltages of current shunts) by the used digital power meter are equal to ±0.2° for 50 Hz and ±1.5° for 5 kHz. In accordance with the law of propagation of the uncertainty of measurements as results from Equations (1) to (5), the combined uncertainties are calculated by taking into consideration the standard deviation of the determination of components U_RSkh_, U_RDkh_, and φ_hk_ for the uniform distribution of errors. Therefore, if the rated secondary current of the tested CT is equal to 5 A, the combined uncertainties type B of the determination of current and phase errors of the transformation of the *hk* harmonic of the distorted current by the measuring systems presented in Figure 2 and Figure 8 are respectively equal to ±0.01%, ±0.01° for its main component of frequency 50 Hz and ±0.05%, ±0.02° for the 100th (5 kHz) higher harmonic. Meanwhile, if the rated secondary current of the tested CT is equal to 1 A, the combined uncertainties type B are respectively equal to ±0.01%, ±0.01° for the 50 Hz frequency main component of distorted current and ±0.03%, ±0.01° for the 100th (5 kHz) higher harmonic. These values were calculated for the distorted current of *hk* harmonic values equal to 0.025\0.05 A, 0.1\0.02 A, 0.5\0.1 A, or 0.6\0.12 A respectively for rms values of the rated secondary current of the tested CT equal to 1\5 A. Under the considered case, values of the phase angle between the *hk* harmonic of voltages from current shunts *R_S_* and *R_D_* were analyzed in 10° steps from 0° to 360° and values of the composite error at the harmonic were equal to 0.1% and 1%. The calculated standard uncertainty type A is negligible as it is significantly lower than 0.1 of uncertainty type B, as results from the averaging from 256 series of measurements. 

**Figure 9:**
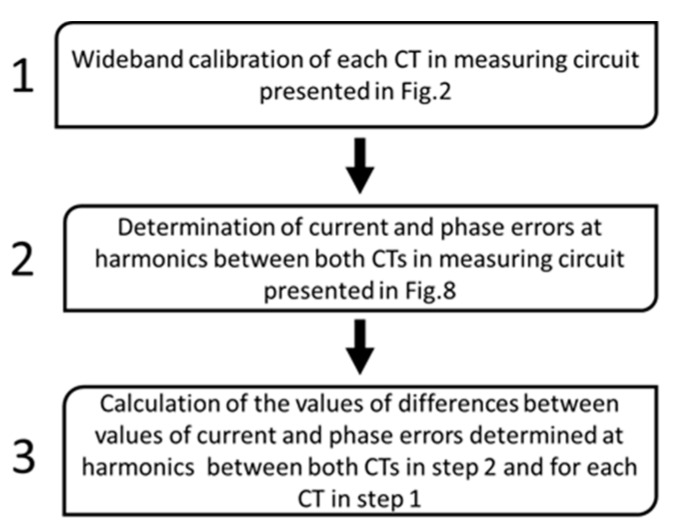
Flowchart of verification of results of the wideband self-calibration of inductive CT.

Verification of the results of the wideband self-calibration of inductive CTs is performed in accordance with the flowchart presented in Figure 9.

In step 1, both inductive window-type CTs are self-calibrated in the measuring circuit presented in Figure 2 in conditions of the ampere-turns obtained by application of additional primary winding. In the next step of the procedure, one of these CTs is used as a reference of primary current for the second CT. The current and phase error of the transformation of higher harmonics of distorted current by this CT are again determined in the measuring circuit presented in Figure 8. In the last step from Equations (10) and (11), the differences are calculated between values of the current and phase errors at harmonics determined in step 2 and during the previous calibration of each CT in step 1.

(10) $$\;\Delta I_{hk\left(diff\right)}=\;\Delta I_{hk\left(D\right)}-\Delta I_{hk\left(AT\_A\right)}-\Delta I_{hk\left(AT\_B\right)}\;$$

(11) $$\delta_{hk\left(diff\right)}=\delta_{hk\left(D\right)}-\delta_{hk\left(AT\_A\right)}-\delta_{hk\left(AT\_B\right)}\;$$

In brackets, the following additional labels are used: AT_ A\AT_B—the values of current and phase errors at harmonics obtained during calibration of each CT in step 1, D—the values of current and phase errors at harmonics measured between both CTs in step 2 of the procedure in Figure 8.

A positive result of verification is achieved when the differences calculated from Equations (10) and (11) are equal to zero. However, due to the fact that the required measurements are performed three times (once for each CT and between them), the considered uncertainties of such a comparison is equal to three times the combined uncertainties type B calculated for current and phase errors. Therefore, if the rated secondary current of the tested CT is equal to 5 A, these values are respectively equal to ±0.03%, ±0.03° for the main 50 Hz component of the distorted current and ±0.12%, ±0.06° for the 100th higher harmonic. Meanwhile, if the rated secondary current of the tested CT is equal to 1 A, the combined uncertainties type B of the verification are respectively equal to ±0.03%, ±0.03° for the 50 Hz frequency main component of the distorted current and ±0.06%, ±0.03° for the 100th higher harmonic.

**Figure 10:**
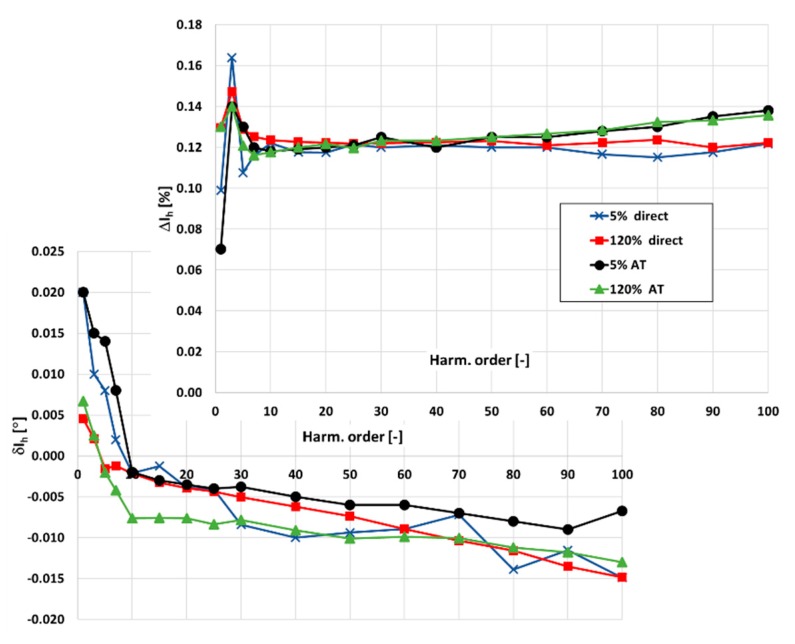
The comparison of the values of current and phase errors at harmonics of the tested CT 300\1 A obtained in relation to the designed CT and in conditions of ampere-turns.

First, the results of wideband calibration of the designed CT 300\5\1 A are verified. Therefore, it is used as the reference of primary current to determine the values of current and phase errors of the transformation of harmonics of the distorted current by the tested CT 300\1 A in the measuring circuit presented in Figure 8. Obtained values in Figure 10 are compared with previously determined values in conditions of ampere-turns. During the tests, the load of the secondary winding is equal to 25% of its rated value. 

In Figure 10, the following notations are used: Direct\AT—values measured with reference CT\in ampere-turns conditions. 

**Figure 11:**
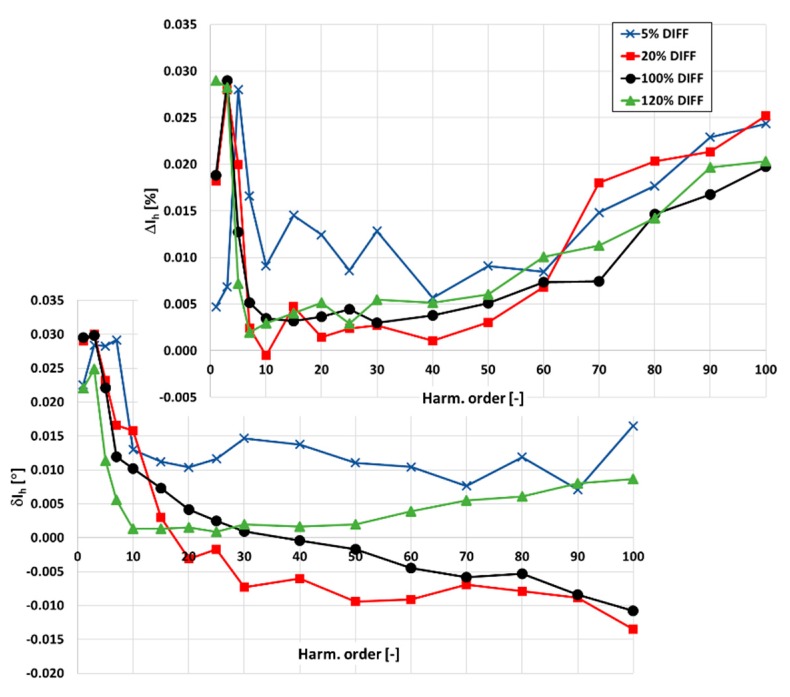
The differences in values of current and phase errors at harmonics measured between CTs 300\1 A and for each CT in the condition of ampere-turns.

Calculated from Equations (10) and (11), differences in the values of current and phase errors at the harmonics measured between CTs 300\1 A and for each CT in the condition of ampere-turns are presented in Figure 11. 

**Figure 12:**
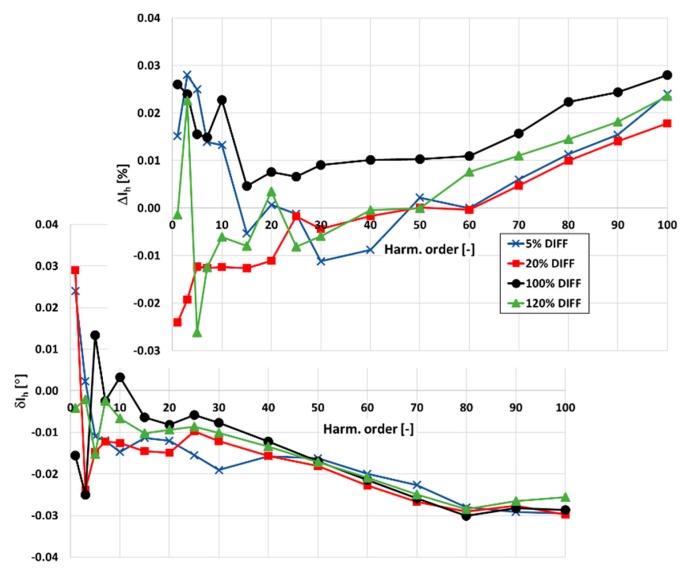
The differences in values of current and phase errors at harmonics measured between CTs 300\5 A and for each CT in the condition of ampere-turns.

The differences in values measured between CTs 300\1 A and for each CT in the condition of ampere-turns do not exceed ±0.03% for the current errors and ±0.03° for the phase errors. These values are within the uncertainties of the verification procedure. Therefore, the values of current and phase errors of the higher harmonic transformation by the designed CT determined in the condition of ampere-turns for the secondary current equal to 1 A are verified. The same procedure is applied for verification of the transformation accuracy of harmonics for the secondary current equal to 5 A (Figure 12). 

In this case, the differences again do not exceed ±0.03% for the current errors and ±0.03° for the phase errors. Therefore, by application of the designed CT as a reference of the primary current in the measuring system presented in Figure 8, its calibration in the measuring system from Figure 2 is verified.

**Figure 13:**
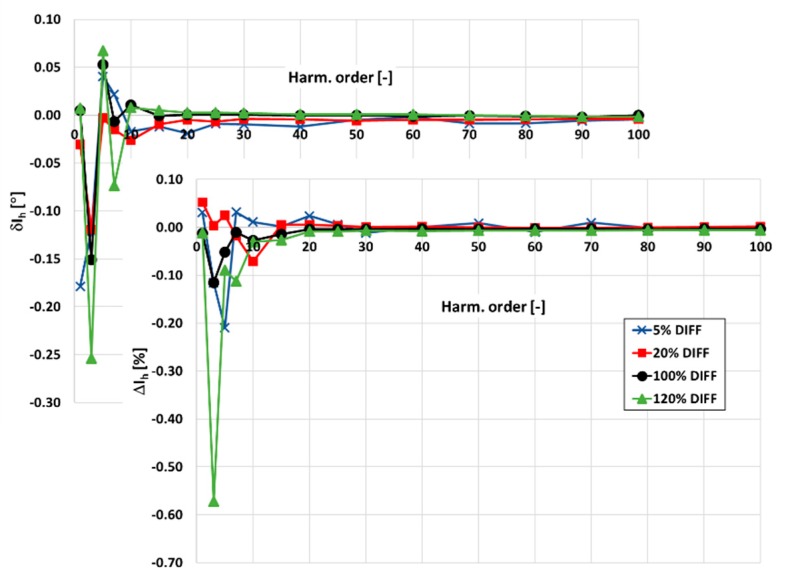
The differences in values of current and phase errors at harmonics measured between class (50 Hz) 0.5 and 0.2 CTs 100\1 A and for each CT in the condition of ampere-turns.

Next, the determined values of the current and phase errors at harmonics obtained for inductive CTs of the rated current ratio equal to 100\1 A are verified. Therefore, in the measuring system presented in Figure 8, current and phase errors at harmonics between CTs class (50 Hz) 0.2 and 0.5 are determined (Figure 13).

The differences in values exceed −0.5% for the current errors and −0.25° for the phase errors. These values are not within the uncertainties of the verification procedure. Therefore, determined in the condition of ampere-turns, values of the current and phase errors of the higher harmonic transformation by both CTs 100\1 A are not verified. This is caused by the high self-distortion of their secondary currents. The rms values of the generated higher harmonics change rapidly with load of the secondary winding. Therefore, a slight change in the value of the connected resistance caused by, e.g., used wires, results in a significant change in their values. Moreover, the values of current and phase errors are still high for the CT of class 0.2, and the changes in their values with the frequency of the transformed higher harmonic are also high (Figure 6). The values of the current error are significantly positive even for conditions of the rated load of the secondary winding. This is due to the decrease in the transformed higher harmonic value of the exciting current with frequency. Therefore, such mass-produced CTs may not be used as a wideband reference of primary current.

**Figure 14:**
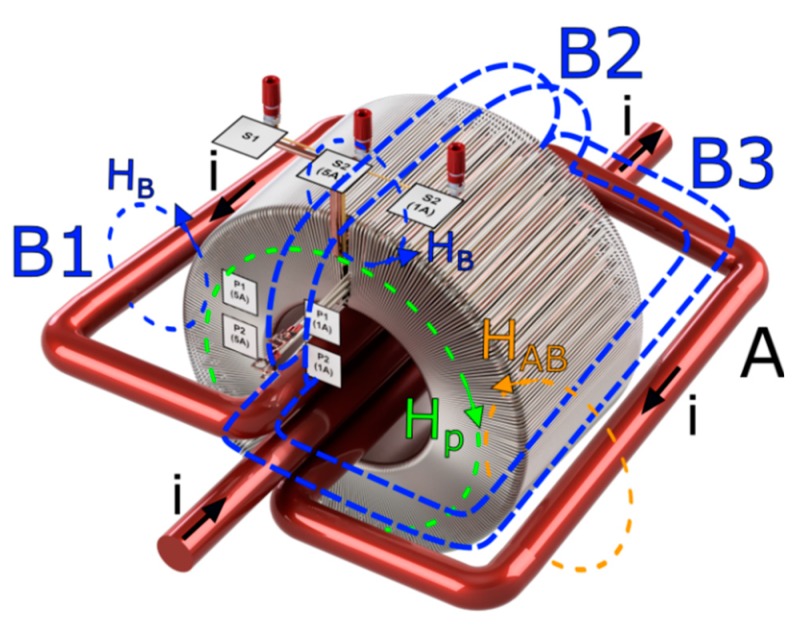
3D view of designed wideband CT with 3 turns of primary wire led through the window of the magnetic core.

In the last stage of the laboratory studies, the increase in the values of the current and phase errors at harmonics caused by returning turns of the primary wire outside of the magnetic core is determined. This results from the increase in the local magnetizing field. During the tests, instead of a single primary wire for a rated current of 300 A, the designed wideband CT is excited by three turns of a wire with the rms value of current equal to 100 A (Figure 14).

**Figure 15:**
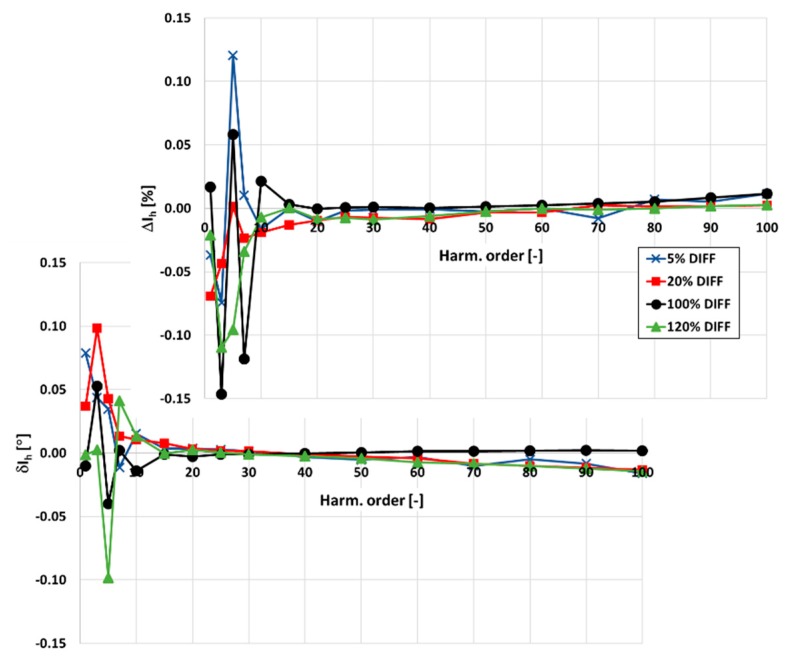
The differences in values of current and phase errors at harmonics determined for designed CT 300\1 A for its excitation by a single primary wire of rated current 300 A and three turns of a wire of rated current 100 A.

Three positions of wire B were tested (B1, B2, and B3). The highest differences in the determined values of current and phase errors at harmonics were found when the wire was placed in position labeled B3. This is due to the fact that the magnetic field strength is added with the magnetic field strength from the wire labeled A. Therefore, the increase in local magnetic field density in the core is the highest. Calculated differences between values of the current and phase errors at harmonics determined for the designed CT 300\1 A (5 A winding is not used) for its excitation by a single primary wire of rated current 300 A and three turns of wire of rated current 100 A are presented in Figure 15. 

An uneven spread of turns of the primary wire on the magnetic core of the designed CT causes an increase in the values of current and phase errors at harmonics from 1st to 10th. Therefore, the values of current and phase errors determined in the condition of ampere-turns are valid only if a single primary wire of rated current 300 A is used. In the cases when the wire is placed in positions B1 or B2 instead of B3, the determined increase in values of the current and phase errors at harmonics from 1st to 10th is lower. However, only if primary wires are evenly spread on the whole surface of magnetic core, the system will be equivalent to excitation with the single primary wire. 

## 4. Conclusions

In this paper, the wideband self-calibration method of window-type inductive CTs in the condition of ampere-turns obtained by application of the additional primary winding is presented. The determined values of current and phase errors of transformation of the harmonics of distorted current are verified by calculation of the differences in values of current and phase errors at the harmonics measured between two CTs and for each CT during calibration. Primary wires must be evenly spread on the whole surface of the magnetic core to obtain equivalent excitation as with a single primary wire. This ensures that values of the current and phase errors of transformation of the harmonics of distorted current are the same in both conditions. Inductive CTs may ensure high accuracy of the transformation of higher harmonics of the distorted primary current only if values of self-generated harmonics into the secondary current are respectively low. However, to limit the distortion of secondary current caused by the nonlinearity of the magnetization characteristic of its magnetic core, it must be significantly oversized in relation to its rated load of the secondary winding. Moreover, if Ni80Fe20 tape is used for the magnetic core of the inductive CT, highest values of the current and phase errors are obtained for transformation of its main component. If turns correction is used to ensure the required accuracy class values of current and phase errors of the transformation of sinusoidal current of the rated frequency, values of the current error at harmonics for the transformation of distorted current may become significantly positive even for conditions of the rated load of the secondary winding. This is due to the decrease with frequency in the transformed higher harmonic values of current associated with the active power losses in the core and the magnetization current. Vectorial diagrams drawn for the transformation of a given harmonic of distorted primary current by inductive wideband CT enables the calculation of values of the corresponding harmonics of current associated with the active power losses in the core and the magnetization current. 

## Figures and Tables

**Table 1:** Limiting values of current and phase errors for 0.05 accuracy class.

I_1_ [%]	ΔI [%]	δ_I_ [°]
5%	±0.15	±0.15
20%	±0.075	±0.075
100%	±0.05	±0.05
120%	±0.05	±0.05

**Table 2:** Values of parameters of the equivalent circuit from Figure 1b for 1st (h1), 5th (h5), and 20th (h20) harmonics of distorted primary current.

Harm. num. Parameter	h1	h5	h20
U_2_ [V]	0.5892	0.0589	0.0589
R_2_ [Ω]	0.0903	0.0903	0.0903
U_R2_ [V]	0.4515	0.0452	0.0452
X_rhk_ [Ω]	0.0090	0.0452	0.1806
U_X2hk_ [V]	0.0452	0.0226	0.0903
L_r_ [mH]	0.0288	0.0288	0.0288
U_μhk_ [V]	1.0420	0.1064	0.1381
ΔI_kh_ [%]	−0.0487	−0.0265	−0.0119
δ_Ikh_ [°]	0.0079	0.0135	0.0038
I^″^_0hk_ [mA]	2.5340	0.1797	0.0679
I_1hk_ [A]	5.0000	0.5000	0.5000
I_2hk_ [A]	4.9975	4.9998	4.9999
I^″^_μhk_ [mA]	0.8035	0.1431	0.0638
L^″^_μhk_ [H]	4.1301	0.4741	0.3452
X^″^_μhk_ [Ω]	1297	744	2165
I^″^_Fehk_ [mA]	2.4030	0.1049	0.0233
R^″^_Fehk_ [Ω]	434	1015	5940
